# Lifestyle interventions to prevent adverse pregnancy outcomes in women at high risk for gestational diabetes mellitus: a randomized controlled trial

**DOI:** 10.3389/fimmu.2023.1191184

**Published:** 2023-08-22

**Authors:** Jiawei Xu, Xuan Lin, Ying Fang, Jing Cui, Zhi Li, Fang Yu, Libin Tian, Hongyan Guo, Xinyan Lu, Jiahao Ding, Lu Ke, Jiahui Wu

**Affiliations:** ^1^ School of Medicine, Wuhan University of Science and Technology, Wuhan, China; ^2^ Department of Endocrinology, CR & WISCO General Hospital Affiliated to Wuhan University of Science and Technology, Wuhan, China

**Keywords:** gestational diabetes mellitus, lifestyle interventions, pregnancy outcomes, prevention, high risk

## Abstract

**Objective:**

To examine the effects of lifestyle interventions, including dietary guidance, health education and weight management, on pregnancy outcomes in women at high risk of gestational diabetes mellitus (GDM).

**Methods:**

Our study included 251 women at high risk of GDM and 128 randomized to lifestyle interventions (dietary guidance, health education, and weight management); One hundred and twenty-three people were randomly assigned to a control group (regular pregnancy check-ups). Counts between groups were compared using either chi-square test or Fisher’s exact test.

**Results:**

Compared with the control group, the risk of GDM was reduced by 46.9% (16.4% vs 30.9%, *P* = 0.007) and the risk of pregnancy induced hypertension (PIH) was reduced by 74.2% (2.3% vs 8.9%, *P* = 0.034) in the intervention group. There were no significant differences in macrosomia, cesarean section, or preterm birth (*P* >0.05).

**Conclusion:**

The lifestyle intervention in this study helped pregnant women to better understand knowledge related to pregnancy, reduce stress and anxiety, and increase intake of adequate prenatal nutrition. This intervention prevented metabolic abnormalities that may occur due to inadequate nutrient intake during pregnancy. In addition, it helped women to control weight gain, maintain appropriate weight gain during pregnancy, and reduce the risk of excessive or insufficient weight gain, ultimately lowering the incidence of GDM and PIH. This highlights the importance of early screening and intervention for high-risk pregnant women.

**Clinical Trial Registration:**

https://www.chictr.org.cn, identifier ChiCTR2300073766.

## 1 Introduction

Gestational diabetes mellitus (GDM) is a carbohydrate intolerance that is diagnosed for the first time during pregnancy (1). It is a common complication during pregnancy because obesity and aging are risk factors (2). With the prevalence of obesity and a sedentary lifestyle, the majority of GDM occurs among women of childbearing age worldwide. According to the 2021 International Diabetes Federation (IDF) Diabetes Map, GDM affects 14.0% of pregnancies worldwide (3). GDM is a severe public health issue with significant short- and long-term adverse health outcomes for mothers and offspring (4–6). GDM is a common cause of cesarean section (CS), preeclampsia (7), and childbirth trauma (8), and it can also result in macrosomia (9). GDM also has long-term effects, including an increased risk of cardiovascular disease in the mother (6, 10) and future type 2 diabetes mellitus (T2DM) and GDM in the fetus (11, 12), with significant financial and health burdens (13). Women with a history of GDM also have a nearly 10-fold increased risk of developing T2DM compared to normoglycemic women. There are no consistent findings regarding the prevention of GDM, and although several trials have been conducted to address the efficacy of lifestyle interventions on the risk of GDM, the results of randomized controlled trials and meta-analyses have varied. The Finnish Gestational Diabetes Prevention Study (RADIEL) showed that lifestyle interventions (providing counseling on diet, physical activity, and weight control) for high-risk pregnant women reduced the incidence of GDM by 39.0% (14). A systematic evaluation and meta-analysis of randomized controlled trials on the relationship between prenatal diet and physical activity interventions and pregnancy outcomes reported that diet and physical activity interventions were associated with a reduced risk of GDM (15). Some studies have found improved glucose tolerance in the intervention group (healthy diet or physical activity), but the risk of GDM did not decrease (16, 17). A meta-analysis conducted in 2015 showed that dietary interventions reduced the incidence of GDM by 33% (18). There is no consensus on the prevention of GDM, and there is a lack of research evidence to guide clinical practice in the prevention of GDM. Therefore, this study aimed to investigate the impact of lifestyle interventions, including dietary guidance, health education, and weight management, on pregnancy outcomes in high-risk pregnant women with GDM. The purpose of this randomized controlled trial was to reduce the incidence of GDM and other adverse pregnancy outcomes in pregnant women at high risk for GDM.

## 2 Materials and methods

### 2.1 Ethical approval and participation consent

This study met the criteria established by the Declaration of Helsinki and was approved by the local ethics committee, the ethics committee of China Resources WISCO General Hospital affiliated to Wuhan University of Science and Technology (registration number HRWGZYY0002). This study was registered with the Chinese Clinical Trial Registry (grant no. ChiCTR2300073766). Participants signed an informed consent form and were informed that the study was designed to promote maternal and fetal health, but were not informed of the primary objectives of the study. The public access link is https://www.chictr.org.cn/.

### 2.2 Sample size calculation

To detect differences in the incidence of GDM between the intervention group (with an expected incidence rate of 15%) and the control group (with an expected incidence rate of 30%), a sample size of approximately 240 pregnant women was calculated with an α level of 0.05 and power of 80%.

### 2.3 General information

From December 2020 to February 2022, 251 pregnant women were recruited in Qingshan District, Wuhan, China. They were assigned to either the intervention group and control groups using a random number table method.

### 2.4 Inclusion and exclusion criteria

#### 2.4.1 Inclusion criteria

Include pregnant women aged 20 years or older and before 20 weeks gestation with one or more risk factors for GDM, such as age ≥ 35 years (19), pre-pregnancy body mass index (BMI) ≥ 25 kg/m^2^ (20), family history of first-degree diabetes (21), history of polycystic ovary syndrome (PCOS) (22), GDM (23), and history of adverse births such as macrosomia, miscarriage, and preterm birth (PTB) (24–26).

#### 2.4.2 Exclusion criteria

Include T2DM, hypertension, hypothyroidism, multiple pregnancies, severely limited food choices, failure to guarantee the number of prenatal check-ups, presence of severe mental disorders, communication disorders, history of antipsychotic use, and history of adverse lifestyle such as smoking, alcohol consumption, and toxic exposure in the past 3 months.

### 2.5 Interventions

A medical team established a WeChat group consisting of doctors, nurses, psychologists, and nutritionists. We invited pregnant women from the experimental group to join the group and provided them with health education, answered their questions in the group, and regularly shared knowledge about healthy pregnancy, such as food choices and weight management. While educating pregnant women, they conveyed pregnancy precautions, helped them eliminate negative emotions, maintain a positive attitude, and cultivate a healthy lifestyle. Additionally, we provided a series of lectures for pregnant women, including “Pregnancy weight management,” “Nutrition and dietary recommendations for pregnant women,” “Prenatal nutrition intervention,” “Blood sugar management during pregnancy diabetes,” and “1000 Days for Health and Nutrition in Early Life.” The pregnant women were also encouraged to keep a food diary and share their experiences in the group. At the time of enrollment, (24-28 weeks and 38-42 weeks), the dietitian performed three nutritional assessments using the 24-hour dietary review method to evaluate the dietary intake status of the pregnant women. Nutritional recommendations were individualized and could be given through the food exchange method, taking into account the preferences and dietary needs of the pregnant women without exceeding the total energy intake. The pregnant women were encouraged to eat a balanced diet with vegetables, fruits, high-fiber whole grain products, low-fat dairy products, increased legumes, nuts and other plant proteins, and other plant proteins while avoiding foods rich in sugar and saturated fatty acids. Reminded pregnant women with excessive or insufficient gestational weight gain (GWG) based on the range of GWG recommended by the 2009 Institute of Medicine (IOM) guidelines (27). The recommended GWG amounts in the IOM guidelines were 12.5-18.0 kg, 11.5-16.0 kg, 7.0-11.5 kg, and 5.0-9.0 kg for pre-pregnancy BMI classification of underweight (BMI < 18.5 kg/m^2^), normal weight (18.5 kg/m^2^ ≤ BMI ≤ 24.9 kg/m^2^), overweight (25.0 kg/m^2^ ≤ BMI ≤ 29.9 kg/m^2^), and obese (BMI ≥ 30.0 kg/m^2^) in women.

### 2.6 Research objectives

Differences in the incidence of GDM and adverse outcomes of interest were observed between the two groups of pregnant women. GDM was defined as one or more pathological glucose values on a 75 g 2-hour oral glucose tolerance test (OGTT) during pregnancy. The diagnostic criteria were as follows: fasting plasma glucose (FPG) ≥ 5.1 mmol/L, 1-hour value ≥ 10.0 mmol/L, 2-hour value ≥ 8.5 mmol/L. Macrosomia was defined as a birth weight of more than 4000 g. PTB was defined as delivery at 28 weeks of gestation but before 37 weeks. Pregnancy Induced Hypertension (PIH) was described as elevated blood pressure (systolic blood pressure ≥ 140 mmHg and/or diastolic blood pressure ≥ 90 mmHg) after 20 weeks of gestation.

### 2.7 Data collection

At the time of maternal enrollment, the general profile of the pregnant women was collected, including age, height, pre-pregnancy weight, gestational week, and GDM risk factors. Pregnant women underwent an OGTT at 24-28 weeks of gestation to determine the presence of GDM. The incidence of maternal and neonatal adverse outcomes, including PIH, PTB, macrosomia, and CS, was recorded after the completion of pregnancy.

### 2.8 Statistical analysis

Count data can be described using frequencies and percentages. For normally distributed continuous data, the mean ± standard deviation (x ± s) is used to describe the data, and between-group comparisons are made using independent-samples t-test. For between-group comparisons of count data, the chi-square test is used. If the data are skewed, the median and interquartile range are used to describe the data, and non-parametric tests are used for comparisons. The data were analyzed by SPSS version 26.0 for Windows (SPSS Inc., Chicago, IL, USA). A value of *P*<0.05 was considered significant. All subjects were included in the statistical analysis, regardless of whether they received the assigned intervention, and were analyzed in the original subgroup.

## 3 Results

**Figure 1:**
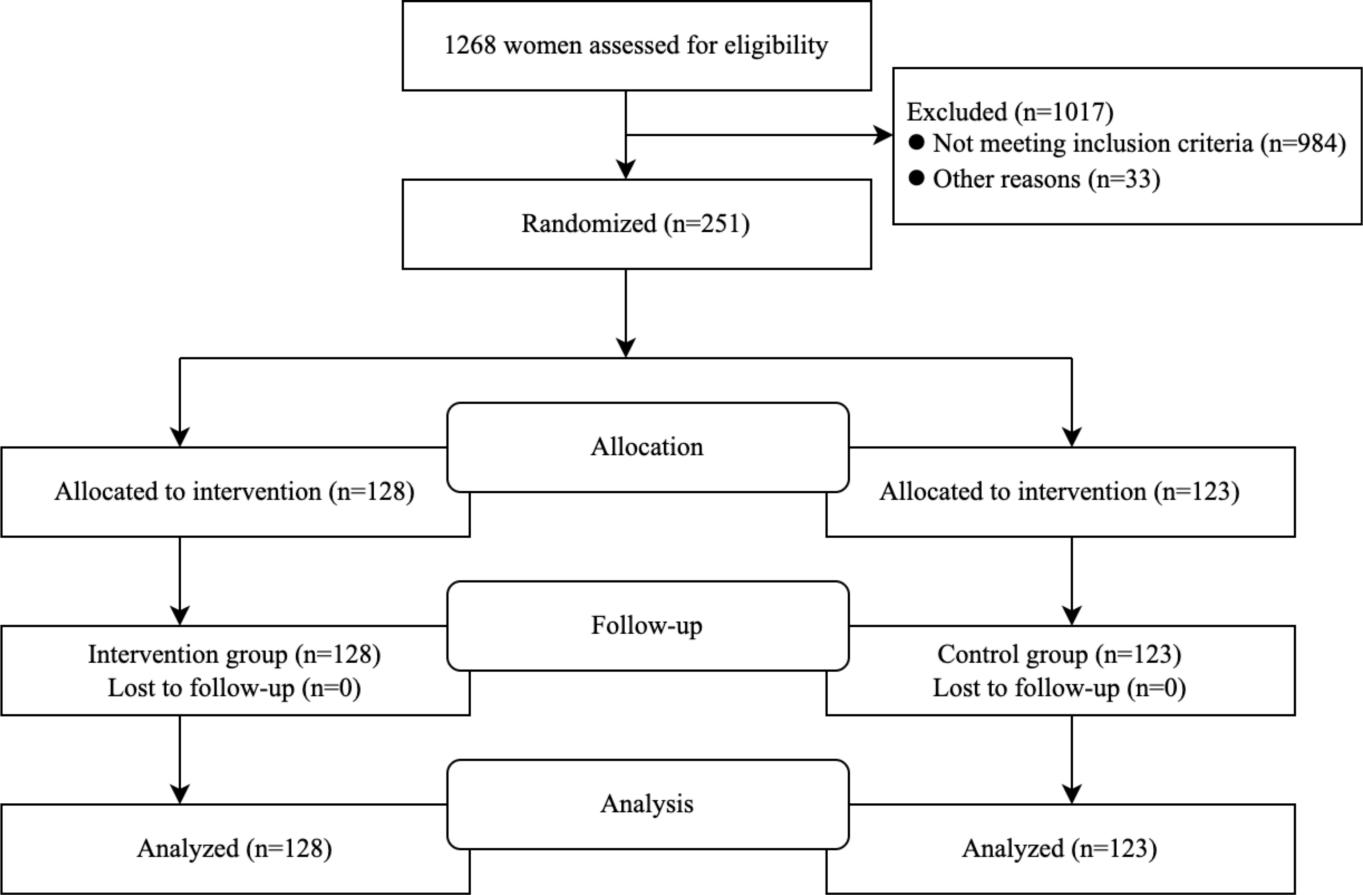
Flowchart.

Of the 1268 pregnant women who underwent eligibility assessment, 984 were excluded because they did not have risk factors for GDM, 6 were excluded due to severely restricted selection criteria, and 27 excluded because they could not guarantee the necessary frequency of obstetric examinations. Eventually, 251 subjects were enrolled in the study; 128 (50.9%) were randomly assigned to the trial group, and 123 (49.1%) were randomly assigned to the control group. The flow chart is shown in **Figure 1**. The baseline characteristics of the participants are shown in **Table 1**. Observations from **Table 2** reveal that in the underweight subgroup, CS was observed more frequently in the higher GWG group. On the other hand, in the overweight subgroup and obese subgroup, the rate of adverse outcomes was lower in the low GWG group when compared to the high GWG group. Observations from **Table 3** indicate that in the GWG too low subgroup, the rate of adverse outcomes was relatively low, and there were no cases of PIH, PTB, and Macrosomia. Similarly, when comparing the underweight subgroup and the normal weight subgroup, the overweight subgroup and obese subgroup showed a higher rate of adverse outcomes in most cases.

**Table 1:** Participant baseline characteristics.

	Intervention group (n=128)	Control group (n=123)	Total (n=251)
Age, years Gestational Week	31.2 ± 3.5 14.1 ± 2.4	31.4 ± 3.6 14.0 ± 2.3	31.3 ± 3.5 14.0 ± 2.4
Age ≥ 35 years old, n (%)	27(21.1)	26(21.1)	53(21.1)
Pre-pregnancy BMI range Pre-pregnancy BMI, kg/m^2^ BMI ≤ 18.5, n (%)	24.5 ± 4.0 8(6.3)	24.4 ± 4.0 8(6.5)	24.5 ± 4.0 16(6.4)
18.5<BMI<25, n (%)	65(50.8)	63(51.2)	128(51.0)
25≤BMI ≤ 30, n (%) BMI>30, n (%)	40(31.3) 15(11.7)	38(30.9) 14(12.2)	78(31.1) 29(11.6)
History of PCOS, n (%) Miscarriage history, n (%) History of PTB, n (%) Birth history of a large child, n (%) Family history of diabetes, n (%) History of GDM, n (%) Race The Han nationality, n (%) Others, n (%)	9(7.0) 18(14.1) 6(4.7) 3(2.3) 30(23.4) 9(7.0) 127(99.2) 1(0.8)	8(6.5) 17(13.8) 7(5.7) 3(2.4) 30(24.4) 9(7.3) 122(99.2) 1(0.8)	17(6.8) 35(13.9) 13(5.2) 6(2.4) 60(23.9) 18(7.2) 249(99.2) 2(0.8)

**Table 2:** Comparison of pregnancy outcomes in the intervention group according to IOM recommendations for GWG too low, normal and too many pregnant women.

Group	n	GDM, n (%)	CS, n (%)	PIH, n (%)	PTB, n (%)	Macrosomia, n (%)
Underweight reorganization	8					
GWG too low group	0	0(0.0)	0(0.0)	0(0.0)	0(0.0)	0(0.0)
GWG normal group	6	0(0.0)	0(0.0)	0(0.0)	0(0.0)	0(0.0)
GWG over multiple groups	2	0(0.0)	2(100.0)	0(0.0)	0(0.0)	0(0.0)
Normal weight reorganization	65					
GWG too low group	5	0(0.0)	1(20.0)	0(0.0)	0(0.0)	0(0.0)
GWG normal group	24	3(12.5)	7(29.2)	0(0.0)	0(0.0)	0(0.0)
GWG over multiple groups	36	5(13.9)	26(72.2)	1(1.5)	0(0.0)	2(3.1)
Overweight reorganization	40					
GWG too low group	1	0(0.0)	0(0.0)	0(0.0)	0(0.0)	0(0.0)
GWG normal group	19	2(10.5)	9(47.4)	0(0.0)	1(5.3)	1(5.3)
GWG over multiple groups	20	4(20.0)	12(60.0)	1(2.5)	0(0.0)	4(20.0)
Obese reorganization	15					
GWG too low group	0	0(0.0)	0(0.0)	0(0.0)	0(0.0)	0(0.0)
GWG normal group	6	2(33.3)	3(50.0)	1(16.7)	0(0.0)	1(16.7)
GWG over multiple groups	9	5(55.6)	6(66.7)	0(0.0)	2(22.2)	4(44.5)
Total	128	21(16.4)	66(51.6)	3(2.3)	3(2.3)	12(9.4)

**Table 3:** Comparison of pregnancy outcomes in the control group according to IOM recommendations for GWG too low, normal and too many pregnant women.

Group	n	GDM, n (%)	CS, n (%)	PIH, n (%)	PTB, n (%)	Macrosomia, n (%)
Underweight reorganization	8					
GWG too low group	1	0(0.0)	0(0.0)	0(0.0)	0(0.0)	0(0.0)
GWG normal group	4	1(25.0)	1(25.0)	0(0.0)	0(0.0)	0(0.0)
GWG over multiple groups	3	0(0.0)	2(66.7)	0(0.0)	0(0.0)	0(0.0)
Normal weight reorganization	63					
GWG too low group	7	2(28.6)	2(28.6)	0(0.0)	2(28.6)	0(0.0)
GWG normal group	25	6(24.0)	10(40.0)	0(0.0)	0(0.0)	0(0.0)
GWG over multiple groups	31	7(22.6)	17(54.8)	1(1.6)	0(0.0)	0(0.0)
Overweight reorganization	38					
GWG too low group	2	0(0.0)	1(50.0)	0(0.0)	0(0.0)	0(0.0)
GWG normal group	13	5(38.5)	8(61.5)	2(15.4)	1(7.7)	3(23.1)
GWG over multiple groups	23	11(47.8)	17(73.9)	3(13.0)	2(8.7)	4(17.4)
Obese reorganization	14					
GWG too low group	1	0(0.0)	1(0.0)	0(0.0)	0(0.0)	0(0.0)
GWG normal group	1	0(0.0)	1(100.0)	0(0.0)	0(0.0)	0(0.0)
GWG over multiple groups	12	6(50.0)	7(58.3)	4(33.3)	4(33.3)	5(41.7)
Total	123	38(30.9)	67(54.5)	10(8.1)	9(7.3)	12(9.8)

As shown in **Table 1**, the intervention group of pregnant women had an age range of 24-39 years (mean 31.2 ± 3.5 years) and a gestational age range of 9-19 weeks (mean 14.1 ± 2.4 weeks) at enrollment. The pre-pregnancy BMI range was 24.5 ± 4.0 kg/m^2^. The control group of pregnant women had an age range of 22-39 years (mean 31.4 ± 3.6 years) and a gestational age range of 9-19 weeks (mean 14.0 ± 2.3 weeks) at enrollment. The pre-pregnancy BMI range was 24.4 ± 4.0 kg/m^2^. The proportion of high-risk factors among the 251 pregnant women participating in the study was as follows: BMI before pregnancy≥ 25kg/m^2^ was 42.6%, family history of diabetes was 23.9%, age ≥ 35 years was 21.1%, history of miscarriage was 13.9%, and history of GDM was 7. 2%, history of PCOS 6.8%, history of PTB 5.2%, history of macrosomia 2.4%. As shown in **Table 4**, the risk of GDM was reduced by 46.9% (16.4% vs 30.9%, *P* = 0.007) and the risk of PIH by 74.2% (2.3% vs 8.9%, *P* = 0.034) in the intervention group compared with complications in the two groups. There were no significant differences between CS (*P* = 0.644), PTB (*P* = 0.065) and macrosomia (*P* = 0.918).

**Table 4:** Pregnancy outcomes were compared between the two groups.

Outcome	Intervention group (n=128)	Control group (n=123)	*P*-value
GDM, n (%)	21(16.4)	38(30.9)	0.644
CS, n (%)	66(51.6)	67(54.5)	0.644
PIH, n (%)	3(2.3)	10(8.9)	0.034*
PTB, n (%)	3(2.3)	4(3.3)	0.065
Macrosomia, n (%)	12(9.4)	12(9.8)	0.918

P*: the difference is statistically significant.

## 4 Discussion

This study successfully reduced the incidence of GDM and PIH through health education, nutritional intervention and weight management for pregnant women at high risk of GDM, and provided a reliable basis for promoting lifestyle interventions in high-risk groups of GDM in the future. However, due to the limitation of study time, the follow-up time of this study was not long enough to observe the longer-term effects of lifestyle interventions on mothers and offspring. It is well-established in previous studies that T2DM can be prevented by lifestyle modification in high-risk individuals (28, 29). Our study aimed to determine whether lifestyle interventions for women at high risk of GDM reduce adverse pregnancy outcomes, with the aim of reducing the incidence of GDM and other adverse pregnancy outcomes. We observed a 46.9% (16.4% vs 30.9%, *P*=0.007) lower risk of GDM and a 74.2% lower risk of PIH (2.3% vs 8.9%, *P*=0.034) in the intervention group compared with the control group. We found that lifestyle interventions reduced the incidence of GDM and PIH, consistent with the results of a meta-analysis (15, 30), which, unlike another meta-analysis (31), which included 23 studies of diet and exercise interventions for the prevention of GDM, found that the risk of gestational diabetes appeared to be lower in the diet and exercise intervention group than in the standard group (RR 0.85, 95% CI: 0.71 to 1.01), probably because the meta-analysis did not take into account the timing and intensity of interventions, which are likely to be important factors of benefit, most studies were initiated in the second or third trimester, missing opportunities to adopt appropriate behaviors in or before pregnancy to prevent GDM, shorter interventions limited the time to improvement of modifiable risk factors, and some studies had ‘mild’ interventions that were insufficient to reduce GDM. Our hypothesized was that initiating lifestyle interventions in the first trimester would allow participants more time to adopt healthy behaviors and lead to better pregnancy outcomes. Previous research has shown that healthy behaviors before pregnancy can have a significant impact on the health of both mother and child (32). Accordingly, it is recommended that women begin adopting healthy lifestyle habits before pregnancy to improve their chances of a healthy pregnancy and reduce the risk of complications. While the incidence of PTB in the intervention group was lower than that in the control group in this study, the difference was not statistically significant (*P* = 0.065), possibly due to the limited sample size. Therefore, increasing the sample size in the future may help to determine whether the observe trend holds. It seems that in this study, the decision to have a CS delivery was mainly based on the preferences of pregnant women and their families, and not necessarily related to the intervention. The hospital advised normal delivery for pregnant women who were capable of it, but ultimately the decision was up to the pregnant woman and her family. It is possible that misconceptions about the benefits and drawbacks of CS delivery, as well as the popularity of painless delivery, may have influenced this decision. Additionally, the duration of normal delivery is often unpredictable, while CS may take less time, which could also be a factor in the decision. The failure to reduce the incidence of large numbers of children in the intervention group as predicted may have resulted in a more effective intervention due to the absence of an increased exercise intervention.

## 5 Conclusions

This study significantly reduced the incidence of GDM and PIH through health education, nutritional intervention and weight management for pregnant women at high risk of GDM, and provided a reliable basis for promoting lifestyle interventions in high-risk groups of GDM in the future. The long-term effects of GDM on patients and their offspring cannot be ignored, but due to the limitation of study time, the follow-up time of this study is not long enough to see the longer-term effect of lifestyle interventions, and the follow-up time needs to be extended to further observe the long-term effects of lifestyle interventions on mothers and offspring.

## Data availability statement

The original contributions presented in the study are included in the article/supplementary material. Further inquiries can be directed to the corresponding author.

## Ethics statement

The studies involving humans were approved by the ethics committee of China Resources WISCO General Hospital of Wuhan University of Science and Technology. The studies were conducted in accordance with the local legislation and institutional requirements. Written informed consent for participation in this study was provided by the participants' legal guardians/next of kin. Written informed consent was obtained from the individual(s) for the publication of any potentially identifiable images or data included in this article.

## Author contributions

XLi helped with study design, data analysis, and statistical analysis. JX and YF performed data analysis, statistical analysis, manuscript writing, and literature search. XLi and YF provided nutritional assessment and guidance for pregnant women, and LT provided weight advice for pregnant women. XLi, YF, JC, ZL, FY, and LT answered questions for pregnant women. JX and YF contributed equally to this work and shared the first authorship. JX, HG, XYL, JD, LK, and JW participated in the data collection and collation. All authors contributed to the article and approved the submitted version.
